# Introducing StatHand: A Cross-Platform Mobile Application to Support Students’ Statistical Decision Making

**DOI:** 10.3389/fpsyg.2016.00288

**Published:** 2016-02-29

**Authors:** Peter J. Allen, Lynne D. Roberts, Frank D. Baughman, Natalie J. Loxton, Dirk Van Rooy, Adam J. Rock, James Finlay

**Affiliations:** ^1^School of Psychology and Speech Pathology, Curtin UniversityPerth, WA, Australia; ^2^School of Applied Psychology, Griffith UniversityBrisbane, QLD, Australia; ^3^Research School of Psychology, Australian National UniversityCanberra, ACT, Australia; ^4^School of Behavioural, Cognitive and Social Sciences, University of New EnglandArmidale, NSW, Australia

**Keywords:** statistics, research methods, selection skills, decision tree, teaching and learning, mobile learning, iOS, web application

## Abstract

Although essential to professional competence in psychology, quantitative research methods are a known area of weakness for many undergraduate psychology students. Students find selecting appropriate statistical tests and procedures for different types of research questions, hypotheses and data types particularly challenging, and these skills are not often practiced in class. Decision trees (a type of graphic organizer) are known to facilitate this decision making process, but extant trees have a number of limitations. Furthermore, emerging research suggests that mobile technologies offer many possibilities for facilitating learning. It is within this context that we have developed StatHand, a free cross-platform application designed to support students’ statistical decision making. Developed with the support of the Australian Government Office for Learning and Teaching, StatHand guides users through a series of simple, annotated questions to help them identify a statistical test or procedure appropriate to their circumstances. It further offers the guidance necessary to run these tests and procedures, then interpret and report their results. In this Technology Report we will overview the rationale behind StatHand, before describing the feature set of the application. We will then provide guidelines for integrating StatHand into the research methods curriculum, before concluding by outlining our road map for the ongoing development and evaluation of StatHand.

## Introduction

Quantitative research methods underpin psychological literacy (McGovern et al., 2010; Cranney and Dunn, 2011; Roberts et al., 2015), and are critical to the development of professional competence in psychology. They have featured prominently in undergraduate psychology curricula since the discipline’s formation (Perlman and McCann, 1999; Saville, 2008), and are reflected in the course learning outcomes and graduate attributes specified by accrediting psychology organizations worldwide. For example, the Australian Psychology Accreditation Council [APAC] (2014, p. 35) specify six graduate attributes for an undergraduate psychology program. Two of these, (“understands the principles of scientific method and is able to apply and evaluate basic research methods in psychology” and “demonstrates the capacity to utilize logic, evidence, and psychological science to evaluate claims about, and solve problems regarding, human behavior”), require a solid and flexible understanding of research methods and statistics. The second of five learning goals for an undergraduate psychology course detailed by American Psychological Association Board of Educational Affairs Task Force on Psychology Major Competencies (2013, p. 15) is “scientific inquiry and critical thinking,” which requires “the development of scientific reasoning and problem solving, including effective research methods,” “applying research design principles to drawing conclusions about psychological phenomena” and “designing and executing research plans.” Similar goals or standards are promoted by the British Psychological Society [BPS] (2014) and other accrediting organizations. Collectively, these standards reflect a widely held understanding that an ability to source, read, understand and critically evaluate relevant research literature is a necessary precursor to evidence-based practice in psychology (American Psychological Association Presidential Task Force on Evidence Based Practice, 2006). The vast majority of this literature is based on quantitative research methods (Kidd, 2002; Rennie et al., 2002). It is also widely held that some of the most effective ways of teaching these skills involve engaging students regularly in all aspects of the research process, from the conception of meaningful research questions, through design, analysis, interpretation and reporting (Marek et al., 2004; Wagner et al., 2011; Earley, 2014; Stoloff et al., 2015). Hence, nearly all psychology departments provide multiple opportunities for undergraduate students to conduct original empirical research, either individually or in collaboration with other students or faculty (Kierniesky, 2005; Perlman and McCann, 2005).

Despite their importance, and their prominence throughout psychology curricula, research methods and (particularly) statistics are recognized areas of weakness for many students (Garfield and Ahlgren, 1988; Murtonen and Lehtinen, 2003; Garfield and Ben-Zvi, 2007; Murtonen et al., 2008). Students are known to particularly struggle with the task of selecting appropriate statistical tests and procedures for different types of research questions, hypotheses and data types; an ability which has been referred to as ‘selection skill’ (Ware and Chastain, 1989). To illustrate this point, Gardner and Hudson (1999) presented 21 brief research scenarios to a sample of 23 students and asked them to recall appropriate statistical procedures for as many scenarios as possible within a 45-min period. The scenarios reflected statistical concepts typically found in introductory statistics textbooks and widely used in behavioral science research. Despite most students having completed at least six research methods and statistics units^1^, they overwhelmingly found the task difficult and performed poorly. On average, students managed to read 10.9 scenarios within the allocated time, and answered 25.3% of them correctly. An additional 15.7% of answers were coded as ‘partially correct.’ When Gardner and Hudson questioned the students about how they made their decisions, several explanations for the poor performance emerged. These included students misinterpreting the research scenarios, knowing but being unable to name appropriate statistics, misidentifying the measurement levels (e.g., nominal, ordinal, continuous) of variables, and seizing on misleading keywords and data presentation formats.

When Allen et al. (2016) presented similar research scenarios to undergraduate psychology students, they also found the the task of identifying appropriate statistical tests and procedures particularly challenging. Many were apologetic, and expressed embarrassment at being unable to successfully complete a task they felt they ought to be equipped to accomplish. When prompted to think about the process of selecting a statistical procedure (rather than actually identifying one), they continued to struggle. The processes they described tended to be haphazard and inefficient, and included looking for clues in the wording of scenarios, searching through textbooks, relying on memory or simply guessing. Of those who recognized that a systematic decision making process could be followed; none could identify every factor that would require consideration, and most also focused on irrelevant or peripheral aspects of the scenarios.

When students are asked to recognize (rather than recall) appropriate statistics, their performance appears similarly underwhelming. For example, Ware and Chastain (1989, p. 225) developed an eight-item multiple-choice selection skill test, which they and colleagues believed contained “problems that students should be able to solve after completing [an] introductory statistics course.” When they administered the test to students at the conclusion of such a course, the students answered fewer than 45% of the items correctly. Ware and Chastain (1989, p. 226) attributed this poor performance, at least partially, to a curriculum which taught statistical techniques “one at a time,” and did not emphasize the development of selection skills. A number of other researchers have also recognized that having relatively few opportunities to practice selection skills could account for the difficulties that students experience when placed in situations where they must work out *which statistic* to use (e.g., Quilici and Mayer, 1996, 2002; Lovett and Greenhouse, 2000; Yan and Lavigne, 2014).

Although not many research methods and statistics courses appear to do so, it is possible to train selection skills. For example, when Ware and Chastain (1991) restructured their introductory statistics course to place greater emphasis on when to use various statistics, and less on computational procedures, they observed a significant improvement on their multiple-choice selection skill test. In a more controlled context, Quilici and Mayer (2002) demonstrated that it is possible to train students to focus on the structural (e.g., the nature of the independent and dependent variables, and the relationship between them) rather than surface-level (e.g., topic) features of basic research scenarios, and that doing so improved students’ abilities to correctly categorize scenarios according to how they would be analyzed. After training, students were also better able to generate new scenarios that could be analyzed using the same statistical procedures as existing scenarios. More recently, similar findings were reported by Yan and Lavigne (2014), who observed that providing students with worked examples emphasizing the structural features of simple research scenarios improved students’ performance on subsequent categorization tasks, as well as their ability to identify the structural features defining each category.

Together, these findings suggest that selection skills are underpinned by ‘structural awareness’ (Quilici and Mayer, 2002), which reflects an ability to disregard the surface features of a research scenario, and focus on its structural features and the relations between them. Like the worked examples used by Yan and Lavigne (2014), graphic organizers, particularly decision trees and flow charts, provide a pedagogical tool for systematically focusing attention on these structural features and relations.

## Graphic Organizers

Graphic organizers are known to facilitate the process of selecting appropriate statistical tests and procedures for different types of research questions and data. They focus the user on each structural component of a research scenario, and illustrate their connectedness/differentiation with spatial positioning and lines (Nesbit and Adesope, 2006). The structured nature of graphic organizers can help users organize new information and integrate it with existing knowledge into schemata (Yin, 2012). The grouping of information lessens cognitive load, and thus more working memory can be applied to learning and problem solving (Yin, 2012). Furthermore, graphic organizers encourage both verbal and spatial encoding of new information, thus providing multiple pathways for its later recall (Katayama and Robinson, 2000). Meta-analyses support the efficacy of concept maps, a type of graphic organizer, for increasing student achievement (Horton et al., 1993), knowledge retention and transfer (Nesbit and Adesope, 2006), and learning (Moore and Readence, 1984).

A number of different types of graphic organizers have been created to help students select appropriate statistical analyses, including tip sheets which sort analyses by their defining characteristics (e.g., Twycross and Shields, 2004), and charts which link statistics to common research goals (e.g., Beitz, 1998). However, the organizers which have gained most traction follow decision tree logic, and are designed to guide the user from an initial question (or problem) to an answer or outcome, via a series of choice or decision points. In domains that involve complex rules, procedures, conditions, and multiple candidate solutions, the use of a decision tree can provide a highly organized approach to the process of decision-making. In the domain of statistics, decision-trees to guide statistical decision making have a long history (e.g., Mock, 1972; Fok et al., 1995) and are now commonly included in statistics textbooks (see, for e.g., Tabachnick and Fidell, 2013; Allen et al., 2014). Statistical decision trees differ from other types of graphic organizers in that they are hierarchical and start with a single node before branching off. By following the branches that refer to the key structural details of a research scenario, the user is led to a statistical analysis appropriate to their circumstances (Mertler and Vannatta, 2002). Theoretically, decision trees rest on the idea that knowledge must be organized or structured to be accessible from long-term memory (Schau and Mattern, 1997). Decision trees provide this structure by explicitly highlighting the interconnectedness (and differentiation) between important statistical concepts (Schau and Mattern, 1997; Yin, 2012).

Empirically, there is work illustrating both the objective efficacy of statistical decision trees, as well as their subjective appeal. For example, Carlson et al. (2005; Protsman and Carlson, 2008) demonstrated that decision trees could facilitate significantly faster and more accurate (by a multiple of three) statistical decision-making, compared to more traditional methods of statistical test selection (e.g., by searching through a familiar textbook). The decision tree method was also significantly more popular amongst students than the textbook method (Carlson et al., 2005; Protsman and Carlson, 2008).

Despite their popularity, traditional statistical decision trees also have limitations. First, they are usually limited in scope by the requirement to fit them on a single sheet of paper, or within the pages of a textbook. Consequently, definitions and other information that would make traversing the tree easier are either spatially separated from the tree itself, or completely absent (Koch and Gobell, 1999; Blankenship and Dansereau, 2000). Second, when given to students without accompanying resources (e.g., a textbook) they do not provide sufficient detail to execute and interpret the statistics they help identify. Third, while the complexity and non-linearity of a statistical decision tree may be helpful to experienced users, new users may experience difficulty in fully processing the tree (sometimes referred to as ‘map shock’), and consequently lose the motivation to use it (Blankenship and Dansereau, 2000; Nesbit and Adesope, 2011).

To overcome these limitations, a number of researchers and educators have adapted the traditional decision tree model for digital media. These hypertext systems are typically comprised of a series of interconnected pages or nodes (Unz and Hesse, 1999). Space constraints associated with paper decision trees are removed, and links can be made to external resources that aid learning (Koch and Gobell, 1999). Map shock can be eliminated because the user is only shown a small section of the tree at any given time, reducing its complexity and ability to overwhelm (Blankenship and Dansereau, 2000). However, a hypertext system can provide a disjointed experience, where users become disoriented and lose track of their location within the system. This phenomenon, sometimes referred to as ‘lost in hyperspace’ (Otter and Johnson, 2000), can constrain the novice user’s ability to develop an understanding of how concepts are connected. Despite this limitation, meta-analytic findings support the overall efficacy of hypertext systems in comparison to textual interfaces. In particular, when compared to textual interfaces, graphical map interfaces are associated with more effective (medium to large effect sizes) and efficient (small to medium effect sizes) performance (Chen and Rada, 1996).

Koch and Gobell (1999) adapted paper decision trees for delivery on the world-wide-web, and in doing so were able to also provide users with definitions, links to online resources, and information about how to enter and analyze data in commonly used statistical software. Like Carlson et al. (2005; Protsman and Carlson, 2008), Koch and Gobell (1999) found that students using their online decision tree were better able to identify appropriate statistical tests than students in a comparison condition. Unfortunately, Koch and Gobell’s (1999) website is no longer active. A current example of an online statistical test selection tool is that provided by University of California, Los Angeles (UCLA)‘s Institute for Digital Research and Education at http://www.ats.ucla.edu/stat/mult_pkg/whatstat/default.htm. This site provides a table of statistical tests based on the number and nature of dependent and independent variables, with ‘how to’ links for a range of statistical software. However, the large size of the table (and the use of a table rather than a decision tree format) combined with the limited information provided may contribute to map-shock for inexperienced users.

A range of software for selecting statistical techniques has also been developed. Some software applications currently available (e.g., Subramanian, 2014; Wacharamanotham et al., 2015) automatically select the statistical test for the user without explicitly guiding the user through the steps to make the decision, greatly reducing their pedagogic potential. STestMAP (Eng et al., 2011) is a visual tool that guides students through a systematic process to select a statistical test, but does not appear to be publicly available. Despite their potential benefits, hypertext decision trees and currently available software generally require the user to have a live internet connection.

## Mobile Learning Technologies

Unlike websites and web applications, mobile learning applications can be developed to maintain all (or most) of their functionality in the absence of an internet connection (Kretser et al., 2015). Mobile learning can be defined as “the use of mobile or wireless devices for the purpose of learning while on the move” (Park, 2011, cited in Yu et al., 2014, p. 2126). In the previous decade, the use of mobile learning technologies such as smart devices and mobile applications has increased rapidly, and amongst western higher education students their penetration is near ubiquitous (Stowell, 2011; Murphy et al., 2013; Dahlstrom and Bichsel, 2014; Chen et al., 2015). Their broad appeal is tied to many factors, including portability, enabling the user to access information and resources virtually anywhere and at any time (Jeng et al., 2010), and utility. Increasingly, students prefer to use their own smart devices for learning, and mobile learning applications have been identified as one of the technologies expected to have the biggest impact on education this decade (Martin et al., 2011; Johnson et al., 2012). In the context of teaching research methods and statistics, emerging research suggests that technology assisted examples delivered via mobile applications positively impact on student learning (Harnish et al., 2012).

## Stathand: A Mobile Application to Support Statistical Decision Making

In the previous sections of this paper, we have described how students find statistical test selection difficult, argued that decision trees can facilitate this decision making process, and noted the rapid adoption of smart devices and mobile learning applications in the higher education sector. With these points in mind, we proposed StatHand to the Australian Government Office for Learning and Teaching in 2013. StatHand was described as a cross-platform mobile application that helps users quickly identify appropriate statistical tests and analytic procedures for a wide range of research questions, hypotheses and data types. The proposal, to develop, disseminate and evaluate StatHand, was funded.

The content of StatHand is being developed in two main phases. The first phase, which is now complete, is focused on helping users identify statistical tests and procedures appropriate to a wide range of circumstances. It is freely available in the iOS App Store, and can also be accessed as a fully mobile-compatible web application at https://stathand.net. The second phase, which is currently under development, guides the computation, interpretation and reporting of these tests and procedures.

**FIGURE 1:**
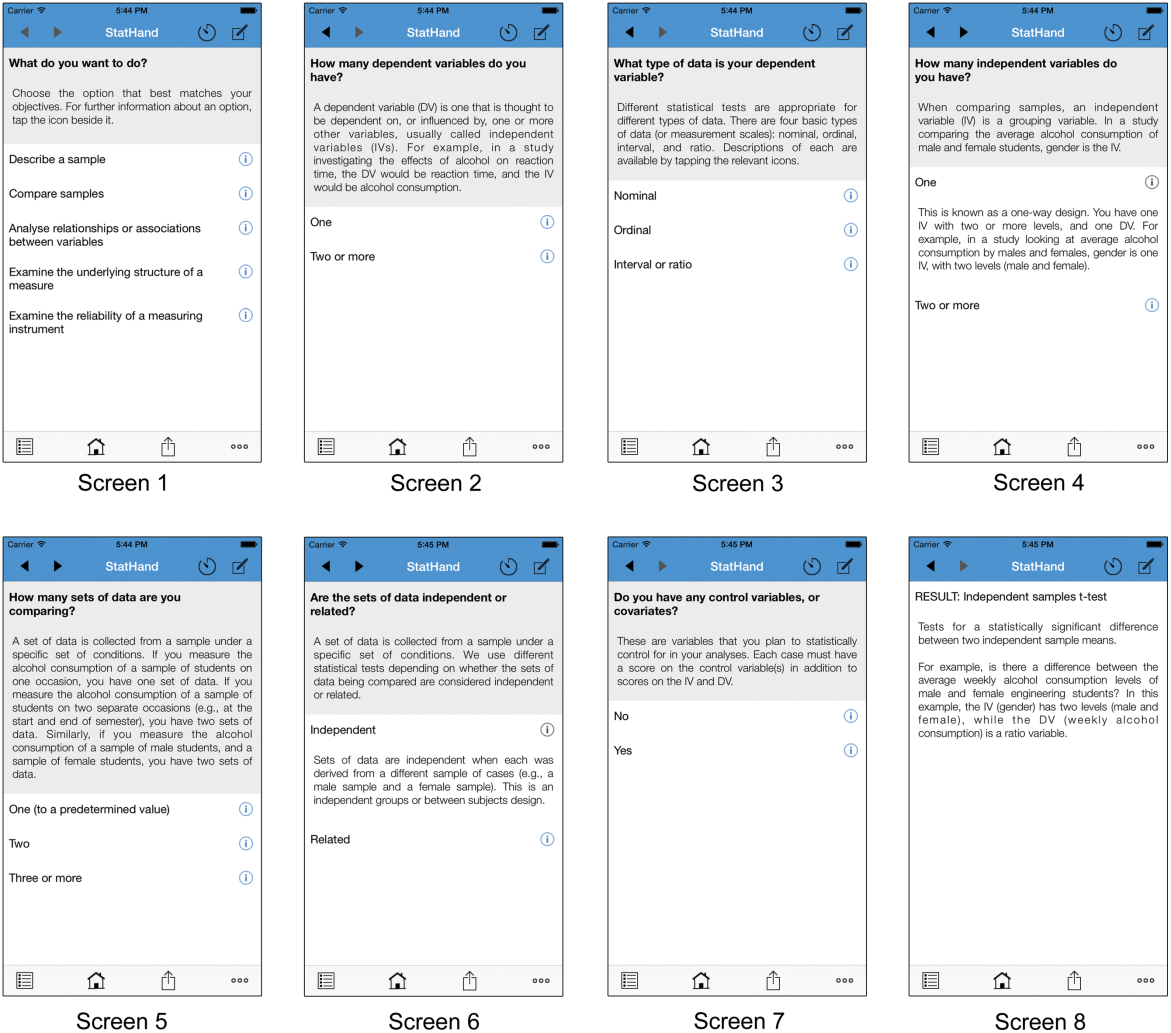
**An illustrative path through the StatHand iOS application on an iPhone 6**. Screens 1–7 depict the decision points that a user would encounter when determining an appropriate statistical test for comparing two independent samples on a continuous dependent measure. Screen 8 depicts the recommended test based on the sequence of decisions made by the user.

The first phase of content is illustrated in **Figure 1**, on the iOS iPhone application. When StatHand is launched (Screen 1), the user is presented with the first of several annotated questions, “what do you want to do?” There are five options available: ‘describe a sample,’ ‘compare samples,’ ‘analyze relationships or associations between variables,’ ‘examine the underlying structure of a measure,’ and ‘examine the reliability of a measuring instrument.’ The statistics, tests and procedures under each of these objectives are listed in **Table 1**. Let’s imagine that we are planning a simple study to examine whether caffeine affects response time. Response time data will be collected for two groups of adults, who will drink either coffee or water immediately prior to testing. The most appropriate option on Screen 1 is ‘compare samples,’ as we wish to compare the performance of the coffee drinkers with that of the water drinkers. After making our first selection, we are presented with a second choice, in which we need to identify the number of dependent variables in the study. A user uncertain about what is meant by ‘dependent variable’ can consult the brief annotation below the question, whereas more experienced users can simply make their selection. Here, we indicate that we have ‘one’ dependent variable (Screen 2), which is measured on an ‘interval or ratio’ scale (Screen 3). Next, we are promoted to consider the number and nature of our independent variable(s). As illustrated in Screens 4 and 6, each option can be expanded for context-specific definitions and examples by tapping on the relevant Information icons. Finally, we are asked to indicate whether or not we have any control variables (Screen 7) which, in the current example, we do not. Having now engaged with each relevant structural feature of our research scenario, we are presented with an appropriate analytic choice (Screen 8). In this case, an independent samples *t*-test.

**Table 1:** The statistics, tests and procedures described in StatHand, grouped by research objective.

Objective	Statistics, tests and procedures described in StatHand
Describe a sample	Bar graph; category count; histogram; interquartile range; Mean; median; mode; pie chart; range; standard deviation; stem-and-leaf plot.
Compare samples	ANCOVA (independent samples and mixed; one way and factorial); ANOVA (independent samples, repeated measures and mixed; one way and factorial); chi-square (goodness of fit and contingencies); Cochran’s *Q* test; Friedman two-way ANOVA; Kruskal–Wallis one-way ANOVA; Mann–Whitney *U* test; McNemar test of change; *t*-test (one sample, independent samples and paired samples); Wilcoxon signed-rank test (one sample and paired samples).
Analyze relationships or associations between variables	Chi-square test of contingencies (with Phi or Cramer’s V); correlation coefficients (point-biserial, rank-biserial, Spearman’s and Pearson’s); eta; linear regression (bivariate and multiple; standard and hierarchical); logistic regression (binary and multinomial; standard and hierarchical); ordinal regression (standard and hierarchical).
Examine the underlying structure of a measure	Confirmatory factor analysis; exploratory factor analysis; principal components analysis.
Examine the reliability of a measuring instrument	Cohen’s kappa; Cronbach’s alpha; intraclass correlation; Kuder–Richardson 20; Weighted kappa.

*The objectives listed correspond with the five options presented to users on the StatHand home screen*.

**FIGURE 2:**
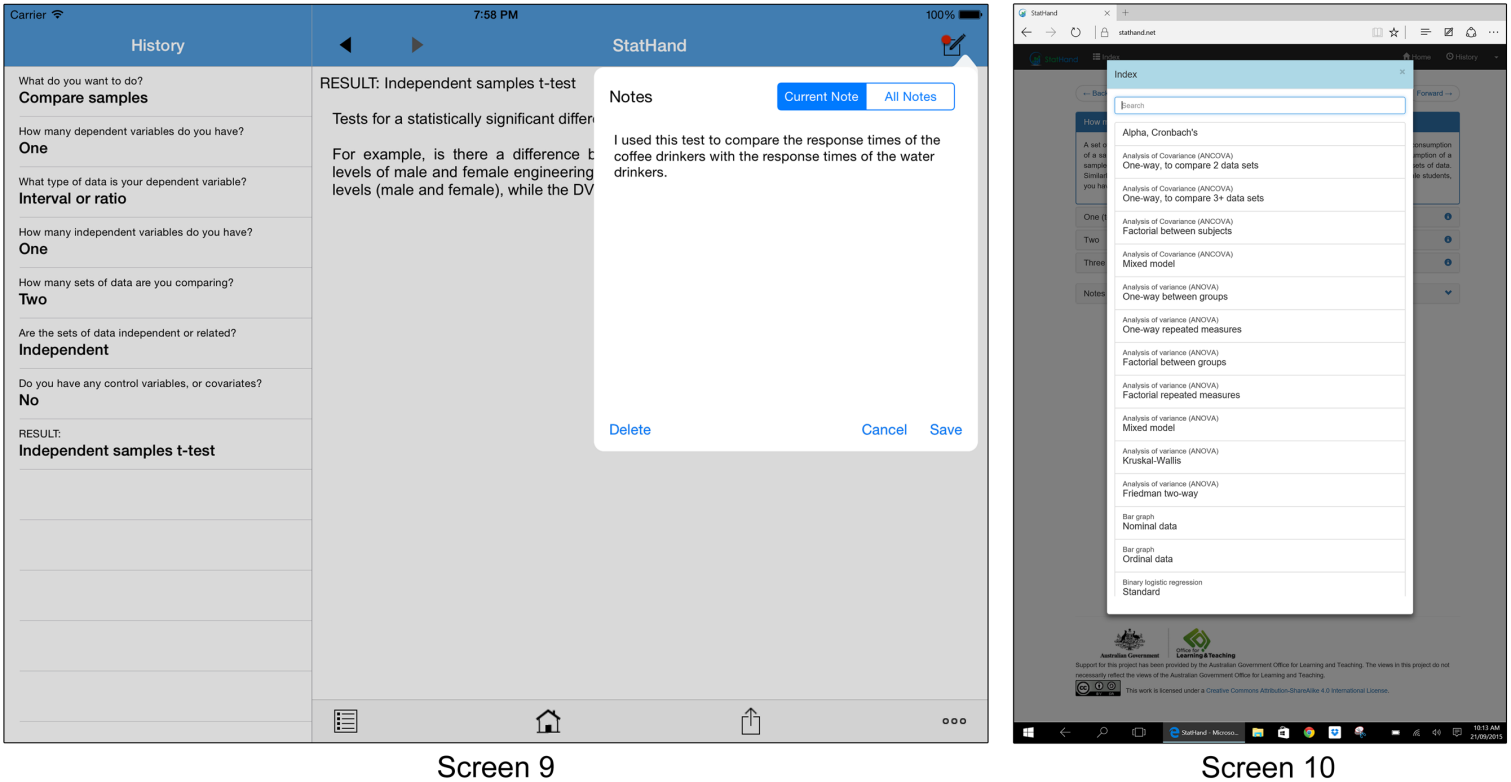
**Screen 9 depicts the StatHand application in landscape mode on an iPad Air 2**. The sequence of decisions leading to an independent samples *t*-test are displayed in the History tool on the left side of the image. Also depicted in Screen 9 is the Notes tool, which can be accessed from any screen by tapping the icon in the upper right corner of the screen. Screen 10 depicts the Index in the StatHand web application, running in Microsoft Edge on a Surface Pro 3.

At any point during the decision making process, a user can review their previous choices using the History tool, as illustrated in Screen 9 of **Figure 2**. This feature allows the user to retrace their steps, and draw stronger connections between their choices and the solutions they reach. Selecting any entry in the History returns the user to the corresponding decision point. Users can also navigate through StatHand using the Back and Forward buttons, or jump directly to a statistic from the searchable Index (illustrated in Screen 10). Also illustrated in Screen 9, **Figure 2** is the Notes tool, with which the user can pin their own annotations to specific pages within the application, or retrieve notes made on other pages. Finally, tapping on the Share icon in the toolbar at the bottom of the screen reveals options to print, email or save the annotated sequence of decisions leading to the current page (including the Notes associated with those decisions). It should be noted that these features work in comparable ways in the web version of StatHand at https://stathand.net, which has been designed for compatibility with any device capable of running a modern web browser.

## Suggestions for Integrating Stathand Into the Research Methods Curriculum

As we’ve observed, many psychology students find the task of selecting appropriate statistics for different research questions, hypotheses and data types challenging (Gardner and Hudson, 1999; Allen et al., 2016). This selection skill (Ware and Chastain, 1989) appears underpinned by structural awareness (Quilici and Mayer, 2002, p. 326); an ability to disregard the surface features of research scenarios, and instead focus on their structural features and the relations between them. Traditional research methods and statistics courses underemphasize these skills, although research suggests that they can be trained (e.g., Quilici and Mayer, 2002; Yan and Lavigne, 2014). Decision trees provide a pedagogic tool for systematically focusing attention on the structural features of research scenarios, as well as the relations between them. StatHand reflects a new breed of interactive decision tree, ready for embedding in existing research methods and statistics curricula. It can be used to provide novel and engaging opportunities to practice selection skills and train students’ structural awareness by systematically sensitizing them to the issues that require consideration before choosing between statistical techniques. Once the second phase content has been deployed, it can further be used as an aid to guide their computation, interpretation and reporting.

Research suggests that integrating technology generally (e.g., Tishkovskaya and Lancaster, 2012; Moreau, 2015), and mobile applications specifically (Harnish et al., 2012) into the research methods and statistics classroom can have pedagogic benefits. However, doing so is not without challenges. Potential barriers to successful integration include the limited confidence of teachers and students when working with new technologies, and differences in learning and teaching styles. Importantly, Lahiri and Moseley (2012, p. 11) cautioned that the use of smart devices as eLearning tools must be underpinned by pedagogical principles and an evidence base, otherwise the use of such tools “might lead to frustration, inequity, shallow learning, and distraction from the main purpose of enhancing learning and making students competent professionals.” Thus, in order to reduce students’ statistics anxiety and enhance students’ selection skills, teachers may wish to consider carefully how to effectively use smart devices as part of the learning process. Yu et al. (2014) stress that smart devices need to be used to extend the reach of teaching. Consequently, “shifting from e-learning to mobile learning implies that instructional designers need to adopt new ways of facilitating learning, not in one way, but using multiple pedagogical strategies, to help people learn whenever they need and wherever they are” (Yu et al., 2014, p. 2132).

StatHand was developed within the theoretical framework of the Unified Theory of Acceptance and Use of Technology (Venkatesh et al., 2003). This theory posits that performance expectancy, effort expectancy, social influence and facilitating conditions are direct determinants of the intention to use a particular technology, with intention and facilitating conditions predictors of actual use. Below we offer some suggestions for embedding StatHand in research methods and statistics courses.

### Demonstrate StatHand at the Outset and Throughout the Course

StatHand is easily and freely accessible via the iOS App Store and online at https://stathand.net. Navigation through the application is intuitive (although brief instructions are available within the application), and largely self-contained, with definitions and examples of all key terms available at a simple tap of an icon. These features increase effort expectancy (defined in terms of ease of use, Venkatesh et al., 2003) Nevertheless, to maximize the application’s perceived utility to students (part of performance expectancy), instructors should devote class time early in the semester to demonstrating how and when to use it. Revisiting StatHand each time a new analysis is introduced will help sensitize students to the similarities and differences between tests vis-à-vis their key structural characteristics (e.g., the key structural difference between the independent samples *t*-test and ANOVA is the number of levels of the independent variable). Such sensitivity is key to structural awareness, and the development of selection skills. Some instructors already use traditional (paper based) decision trees in efforts to achieve this aim. The benefits of transitioning to StatHand include the reduced potential for map-shock or ‘glossing over key decision points,’ the provision of an additional set of examples that students can refer to when seeking to master complex concepts, and the ability for students to save, print or email a record of their sequence of decisions (and annotations associated with those decisions) for later reference. Performance expectancy will increase as students succeed in selecting appropriate statistical techniques using StatHand.

### Link StatHand to Existing Teaching Resources

StatHand can be easily incorporated into existing teaching activities and resources. For example, one of us (NL) created a YouTube screencast demonstrating the use of StatHand and embedded a link to the screencast (along with links to StatHand) in an existing worksheet demonstrating how to perform and interpret a specific statistical procedure. Another of us (PA) regularly uses it in tutorial activities and assessments, where students are presented with a research scenario and data set, and required to generate meaningful hypotheses. StatHand is then used to identify appropriate hypothesis tests, which are conducted and interpreted in the remainder of the class. The linking of StatHand to existing teaching resources combined with the annotated question feature of the StatHand app provide organization and technical infrastructure (facilitating conditions) to support adoption and use. The use of StatHand within existing forums such as discussion boards and social media sites facilitates social influence, particularly if used across multiple courses within the student’s degree.

### Minimize Competition from other Sources

Competition from other sources of interaction when using technology in the classroom can impact on focus. To limit such distractions, students will need to be given clear advice about how to maximize the benefits that can be derived from using learning technologies. At a minimum, this may include recommending turning on ‘airplane’ mode on smart devices, which will prevent them from receiving notifications, and reduce students’ temptation to check emails, browse the web or use social networking applications.

### Use StatHand Consistently and Repeatedly Throughout the Course (and other Related Courses)

When used effectively, StatHand can reinforce information provided by instructors, and offer practical experience in determining appropriate analyses for a variety of different research scenarios. When used consistently through statistics courses, and when statistical decision-making is explicitly assessed, selection skills can be generalized to other research-related courses. As a single application available free on a wide variety of platforms, StatHand can be readily incorporated across multiple courses in statistics and other research-focused courses throughout the psychology undergraduate degree. Over time, students will become increasingly familiar with StatHand, the promotion of its use by multiple instructors will enhance social influence, and both the intention to use, and actual use of StatHand. Its use will be second nature by the time they begin conducting individual (or small group) research projects in their final years of study.

## Future Directions and Conclusion

StatHand is a cross-platform application designed to aid the process of selecting statistical tests and procedures for a wide range of research scenarios. It is currently available in the iOS App Store and at https://stathand.net. StatHand can be easily integrated into existing teaching and learning activities, or used as a base for the development of new activities focused on exploring the circumstances in which different statistics are appropriate.

Content for the second phase of StatHand is currently under development. When incorporated into the iOS and web applications, it will guide users through the computation, interpretation and reporting of each statistic that StatHand helps identify (see **Table 1**). It will also provide advice on testing assumptions and calculating and interpreting effect sizes where appropriate; offer links to additional reputable information about each technique; and highlight controversies and alternative approaches where applicable. Much of this material is being prepared as short videos, developed following evidence-based multimedia learning object design principles (e.g., Clark and Mayer, 2011).

We have also started integrating StatHand into our own research methods and statistics units. This is informing the development of a set of instructors’ resources to complement StatHand. These resources will include a brief rationale for the use of the application as a learning and teaching tool, instructions for using the application, tips for integrating StatHand into undergraduate research methods and statistics classes, and active learning activities that instructors can adapt for their own teaching purposes. The package of activities will include multiple-choice quizzes that instructors can use to assess their students’ abilities to identify appropriate statistical tests and procedures under a wide variety of circumstances. These will be provided in formats suitable for inclusion in worksheets and tests, as well as formats suitable for inclusion in PowerPoint presentations that either do or do not make use of common audience response technologies (e.g., Turning Point Keepad). When available, the StatHand instructors’ resources will be provided freely, on request, to anyone who teaches research methods, statistics and related subjects at recognized higher education institutions.

Dissemination of StatHand is ongoing, and as its user base expands we are collecting usage data that will inform how the application may be optimized to facilitate learning and the decision making process. Additional research projects are experimentally investigating the instructional efficiency of StatHand relative to other common decision making aids (e.g., paper based decision trees and familiar textbooks). Further research will empirically investigate students’ adoption and use of StatHand within the Unified Theory of Acceptance and Use of Technology framework (Venkatesh et al., 2003). Finally, we will soon begin investigating how instructors use StatHand to support the learning and teaching within their own courses. This multi-pronged evaluation approach has two ultimate aims. The first of these is to inform the ongoing development of StatHand. The second is to develop an evidence base and best-practice recommendations to guide its use.

To conclude, in this Technology Report we have provided an overview of StatHand, a free cross-platform mobile application designed to support students’ statistical decision making. Developed with the support of the Australian Government Office for Learning and Teaching, StatHand guides users through a series of simple, annotated questions to help them identify a statistical test or procedure appropriate to their circumstances. In its next release, StatHand will also guide the computation, interpretation and reporting of the tests and procedures it helps users identify. We invite psychology research methods and statistics instructors to contact us about incorporating StatHand into their own classes.

## Author Contributions

PA led the development of the StatHand application, with support and contributions from LR, FB, NL, DVR, and AR. All authors contributed to the preparation of this manuscript.

## Conflict of Interest Statement

The authors declare that the research was conducted in the absence of any commercial or financial relationships that could be construed as a potential conflict of interest.

## Footnotes

1 In Australia, a ‘unit’ is a single subject, typically taken alongside two or three others over a semester. This term is analogous to ‘course’ in the United States.
